# Variation in severe postpartum hemorrhage management: A national vignette-based study

**DOI:** 10.1371/journal.pone.0209074

**Published:** 2018-12-13

**Authors:** Anne Rousseau, Patrick Rozenberg, Elodie Perrodeau, Philippe Ravaud

**Affiliations:** 1 Department of Obstetrics and Gynecology, Poissy-Saint Germain Hospital, Poissy, France; 2 INSERM U1153, METHODS (Méthodes en Évaluation Thérapeutique des Maladies Chroniques) Research Unit. Paris Descartes-Sorbonne Paris Cité University, Paris, France; 3 Research Unit EA 7285, Versailles-St Quentin University, Saint Quentin en Yvelines, France; 4 Assistance Publique-Hôpitaux de Paris, Centre d’Epidémiologie Clinique, Hôpital Hôtel-Dieu, Paris, France; University of Sydney, AUSTRALIA

## Abstract

**Objectives:**

To assess variations in management of severe postpartum hemorrhage: 1) between obstetricians in the same situation 2) by the same obstetrician in different situations.

**Study design:**

A link to a vignette-based survey was emailed to obstetricians of 215 maternity units; the questionnaire asked them to report how they would manage the PPH described in 2 previously validated case-vignettes of different scenarios of severe PPH. Vignette 1 described a typical immediate, severe PPH, and vignette 2 a less typical case of severe but gradual PPH. They were constructed in 3 successive steps and included multiple-choice questions proposing several types of clinical practice options at each step. Variations in PPH were assessed in a descriptive analysis; agreement about management and its timing between vignette 1 and vignette 2 was assessed with the Kappa coefficient.

**Results:**

Analysis of complete responses from 119 (43.4%) obstetricians from 53 (24.6%) maternity units showed delayed or inadequate management in both vignettes. While 82.3% and 83.2% of obstetricians (in vignettes 1 and 2, respectively) would administer oxytocin 15 minutes after PPH diagnosis, only 52.9% and 29.4% would alert other team members. Management by obstetricians of the two vignette situations was inconsistent in terms of choice of treatment and timing of almost all treatments.

**Conclusion:**

Case vignettes demonstrated inadequate management as well as variations in management between obstetricians and in different PPH situations. Protocols or procedures are necessary in all maternity units to reduce the variations in practices that may explain a part of the delay in management that leads to PPH-related maternal mortality and morbidity.

## Introduction

Severe postpartum hemorrhage (PPH) is a leading cause of maternal mortality and morbidity worldwide [1–4] and occurs in around 1% to 2% of deliveries [3,4].

The initial treatment of severe PPH involves medical management, uterine massage, and uterotonic drugs such as oxytocin and prostaglandin. When these first-line treatments fail to control a hemorrhage, intrauterine balloon and/or invasive therapy for postpartum hemorrhage is usually recommended, as shown by similar national guidelines from several countries [5–8]. Intrauterine tamponade is now considered the leading second-line therapy, for it avoids the need for further interventional surgery in most cases [9,10]. Surgical procedures, such as uterine compressive sutures, vascular ligation, and arterial embolization, can be attempted to avoid hysterectomy and have similar effectiveness rates—around 60–80% [11–18].

Management of severe PPH, however, is less well standardized than its prevention. Variations in first-line management occur between and within countries [19,20]. Moreover, reports from confidential enquiries have shown that as many as 67% of the deaths in the United States and 85% of those in France are avoidable, resulting as they have from either delayed or inadequate treatment [21–23].

Furthermore, the lack of comparative effectiveness studies makes it impossible to prefer one of the conservative surgical approaches over any other. The choice and timing of invasive second-line therapies are less standardized and may vary widely between and within countries [18,24–26]. A cohort study in the United Kingdom showed that 25% of PPH were managed with intrauterine tamponade before one of the specific invasive second-line therapies: embolization (8%), vascular ligation (7%), uterine compressive suture (73%), and hysterectomy (9%) [18]. A French cohort study reported that the first-line invasive therapy was arterial embolization for 62% of women, conservative surgery for 26%, and hysterectomy for 12% [25].

Because PPH cases are so heterogeneous, it is difficult to know whether these variations in practices are related to the clinical situation encountered, the professional environment, or the professionals themselves. The existence of variations in PPH management between different situations for the same obstetrician has not yet been investigated.

Clinical vignettes have been widely used to compare quality of clinical care and to assess practice variations across countries, health care systems, specialties, and clinicians [27–29]. The case-vignette method can be used to identify variations in practice and to overcome the limitations due to the variations related to the PPH situation itself. These vignettes thus make it possible to assess variations between obstetricians dealing with the same situation and in two situations for the same obstetrician. In a previous study, dynamic vignettes with several steps proved to be a valid tool that can accurately reflect real practices in such complex emergency situations as severe PPH with second-line therapies [30,31].

The objective of our study was to assess variations in management of PPH: 1) between obstetricians faced with the same situation, and 2) for the same obstetrician in different situations involving PPH.

## Materials and methods

This multicenter cross-sectional study took place from January to June 2014.

Obstetricians received an email with a link to a dedicated website, where they were asked to complete this survey, by responding to questions about how they would manage 2 case-vignettes of severe PPH.

### Survey instrument: Case-vignettes

To validate the use of dynamic case-vignettes describing incidents of severe PPH in several steps, we developed 66 such vignettes, based on documentation in patient files [30]. The study design, details, and findings of this validation study have previously been published [30].

We subsequently used this method to assess care by midwives [31] and now, as we report here, by obstetricians. The 2 vignettes selected were identical for the surveys of midwives and of obstetricians. Experts jointly selected these 2 case-vignettes among the initial 66. One was a typical immediate PPH (Vignette 1), while the other involved a less usual case in which a constant trickle of blood gradually became a PPH (Vignette 2) (see Files in S1 and S2 Files).

All of these vignettes were designed to include 3 successive steps describing a PPH that is worsening even though care is underway. Successive steps also enabled us to assess choices for second-line therapies after the failure of pharmacological treatment to control the bleeding. The vignette began by reporting the medical history, labor, delivery, and PPH; it included a partogram. The next two steps described the response to treatment (postpartum course) over the next two successive 15-minute periods, including a simulated photograph from which blood loss could be assessed [32] and a monitor screen reporting pulse, blood pressure, and SpO_2_. Both reported the same volume of final blood loss: 1.2 L. At each step, the same closed-ended questions were used to ask obstetricians how they would manage the emergency and to obtain details about three general types of management: pharmacologic, non-pharmacologic, and communication/monitoring/investigation. The specialists were to choose none, one, or more actions from a list of choices for each type, repeated at each step:

- oxytocin, misoprostol (prostaglandin E1 analogue), or sulprostone (prostaglandin E2 analogue) for pharmacological management;
- uterine massage, manual uterine examination, cervical examination, perineal repair, intrauterine tamponade, selective arterial embolization, or surgical treatment, including uterine compressive suture, arterial ligation, or hysterectomy, for the non-pharmacological procedures;
- and finally alert an additional obstetrician (senior if available) and an anesthetist, as action for communication, monitoring, and investigation.

Participants could not return to a step to change their response once they had answered all questions at that step.

Appropriate answers were defined by expert consensus according to international guidelines [31]. In step 1, obstetricians were expected to choose oxytocin administration and manual uterine examination; in step 2, sulprostone, and in step 3, a second-line therapy. Finally, alerting other members of the team was expected to have been chosen in one of the three steps.

### Participants

We randomly selected 15 of 35 perinatal networks in France to include about half of all French maternity units. All maternity units here, both public and private, belong to a perinatal network that groups together level-1 (no facilities for nonroutine neonatal care) and level-2 (with a neonatal care unit) units around one or more level-3 units (reference centers with an onsite neonatal intensive care unit). All 215 maternity units of 15 perinatal networks were eligible.

### Survey administration

We sent an email to the supervising obstetricians of all selected perinatal networks, explaining the aim of the survey and asking them to forward by email the link to the survey website to full-time obstetricians of all maternity units of perinatal network. Two gentle email reminders were sent 2 months apart to obstetricians through their supervising obstetricians [33].

### Ethics statement

Our institutional Review Board (Comité de Protection des Personnes Ile de France Paris- XI) approved this study on September 13, 2012, as number 12066.

By clicking on the survey link and completing the questionnaire, obstetricians provided informed consent to participate. Participants were informed about the purpose of the study at the beginning of the study through the email that led them to the study website and by the introduction to the study.

### Role of the funding source

The study sponsor did not participate in the study design, data collection and analysis, decision to publish, or preparation of the manuscript. Authors had full access to all the data and had final responsibility for the decision to submit for publication.

### Statistical analysis

Data are available in Table in S1 Table.

Characteristics of obstetricians and management were described with means and standard deviation (SD) for quantitative variables and with frequencies and percentages for qualitative variables.

Agreement about the steps required for management and their timing (in step 1, 2, or 3) between vignette 1 and vignette 2 was assessed with the Kappa coefficient. Based on the standards outlined by Landis and Koch [34], a Kappa coefficient <0 was considered to be poor agreement, 0–0.20 slight, 0.21–0.40 fair, 0.41–0.60 moderate, 0.61–0.80 substantial, and 0.81–1.0 almost perfect agreement.

All statistical tests were two-sided and P-values<0.05 were considered statistically significant. We used R software, version 3.0.1.

## Results

Among the 215 maternity units approached, 53 (24.6%) participated. Among the 274 full-time obstetricians working in these 53 maternity units, 119 (43.4%) responded to the survey. Table 1 summarizes the characteristics of obstetricians and their maternity units. Our sample showed an over-representation of public maternity hospitals, which accounted for 41 of the 53 participating units (77%), versus 140 of the 215 (65%) maternity units included.

**Table 1:** Characteristics of obstetricians and maternity units.

Obstetricians			n = 119
Age, years	mean (SD)		44.7 (11)
	median [interquartile range]		43 [33.5 ; 55]
Experience > 4 years	n (%)		113 (95.0)
Gender : male	n (%)		58 (48.7)
Maternity unit status	n (%)		
		public university	34 (28.6)
		other public	61 (51.3)
		private	24 (20.1)
Number of births per year	n (%)		
		< 1500	50 (42.0)
		1500≤ b/y <3000	32 (26.9)
		≥ 3000	37 (31.1)

**Fig 1:**
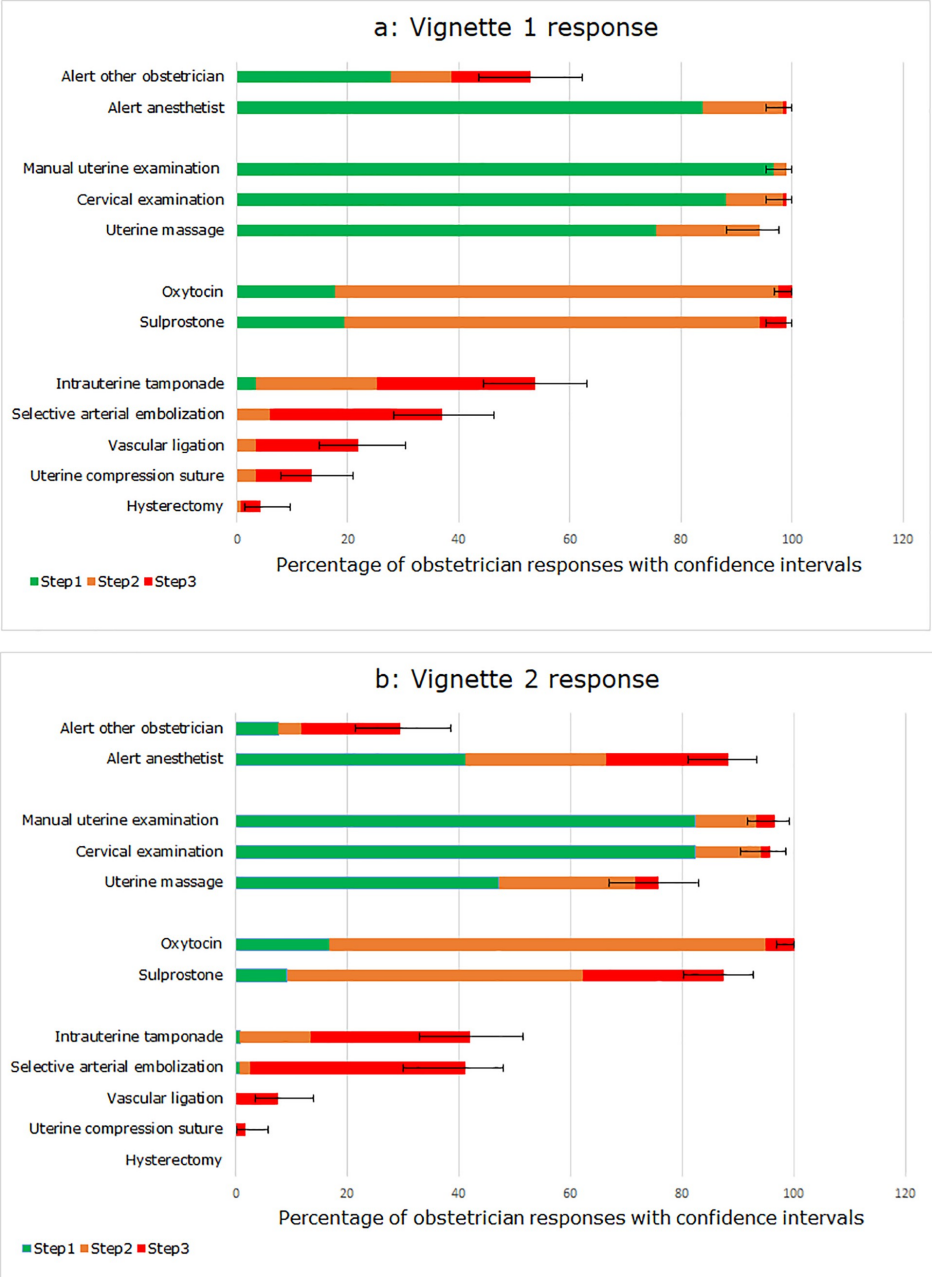
**PPH management for vignette 1 (a) and vignette 2 (b).** This figure summarizes the actions selected by obstetricians at each step for vignette 1 and vignette 2.

Fig 1 summarizes the variations in management actions and their timing among obstetricians faced with the same situation in vignettes 1 and 2, respectively. For example, in vignette 1, 27.7% of obstetricians alerted another obstetrician in step 1 (during the first 15 minutes after diagnosis), 10.9% in step 2 (between 15 and 30 minutes), 14.3% in step 3 (after 30 minutes), while 47.1% did not choose to do so at all.

Fig 1A, corresponding to vignette 1, shows delays in management: 82.3% of obstetricians would not administer oxytocin (first-line uterotonic) until after step 1 (the first 15 minutes after diagnosis); 5.9% would not administer prostaglandin in the first 30 minutes, and 28.6% would not start intrauterine tamponade in step 3 (after 30 minutes). These delays were still more pronounced for vignette 2 (Fig 1B).

Table 2 summarizes the agreement between vignette 1 and vignette 2 for each individual obstetrician for each action. It was slight for most treatments except oxytocin (fair), selective arterial embolization (fair), and intrauterine tamponade (moderate) and thus suggest poor agreement between vignette 1 and vignette 2 for individual obstetricians.

**Table 2:** Agreement about management actions and their timing between vignette 1 and vignette 2.

		Vignette 1	Vignette 2	Kappa coefficient
		n = 119	n = 119	agreement V1-V2
		n (%)	n (%)	
Alert additional obstetrician				0.33 [0.22 ; 0.45]
	step 1	33 (27.7)	9 (7.6)	fair agreement
	step 2	13 (10.9)	5 (4.2)	
	step 3	17 (14.3)	21 (17.6)	
Alert anesthetist				0.14 [0.05 ; 0.23]
	step 1	100 (84.0)	49 (41.2)	slight agreement
	step 2	17 (14.3)	30 (25.2)	
	step 3	1 (0.8)	26 (21.8)	
Manual uterine examination				0.04 [-0.10 ; 0.18]
	step 1	115 (96.6)	98 (82.3)	slight agreement
	step 2	3 (2.5)	13 (10.9)	
	step 3	0	4 (3.4)	
Cervical examination				0.13 [-0.06 ; 0.32]
	step 1	105 (88.2)	98 (82.3)	slight agreement
	step 2	12 (10.1)	14 (11.8)	
	step 3	1 (0.8)	2 (1.7)	
Uterine massage				0.12 [0.01 ; 0.24]
	step 1	90 (75.6)	56 (47.1)	slight agreement
	step 2	22 (18.5)	29 (24.4)	
	step 3	0	5 (4.2)	
Oxytocin				0.27 [0.10 ; 0.44]
	step 1	21 (17.7)	20 (16.8)	fair agreement
	step 2	95 (79.8)	93 (78.1)	
	step 3	3 (2.5)	6 (5.1)	
Prostaglandin				0.09 [-0.02 ; 0.20]
	step 1	23 (19.3)	11 (9.2)	slight agreement
	step 2	89 (74.8)	63 (52.9)	
	step 3	6 (5.0)	30 (25.2)	
Intrauterine tamponade				0.42 [0.29 ; 0.55]
	step 1	4 (3.4)	1 (0.8)	moderate agreement
	step 2	26 (21.8)	15 (12.6)	
	step 3	34 (28.6)	34 (28.6)	
Selective arterial embolization				0.21 [0.05 ; 0.36]
	step 1	0	1 (0.8)	fair agreement
	step 2	7 (5.9)	2 (1.7)	
	step 3	37 (31.1)	46 (38.7)	
Vascular ligation				0.11 [-0.06 ; 0.28]
	step 1	0	0	slight agreement
	step 2	4 (3.4)	0	
	step 3	22 (18.5)	9 (7.6)	
Uterine compressive suture				0.03 [-0.06 ; 0.12]
	step 1	0	0	slight agreement
	step 2	4 (3.4)	0	
	step 3	12 (10.1)	2 (1.7)	
Hysterectomy				0 [-2 10^−7^ ; 2 10^7^]
	step 1	0	0	slight agreement
	step 2	1 (0.8)	0	
	step 3	4 (3.4)	0	

## Discussion

### Main findings

Our study shows important variations in actions to treat PPH and their timing, both between obstetricians faced with the same situation and for two different situations for the same obstetrician. It also showed important delays in treatment that amounted to inadequate management; these were worse in vignette 2, involving gradual PPH with a constant trickle of blood.

### Clinical meaning of the study

The variations we observed in PPH management and the delays or inadequate management sometimes found concerned not only second-line therapies but also initial management.

Variations in invasive second-line therapies are easily explained by their lesser standardization in international clinical guidelines [5–8]. American, British, French, and Canadian guidelines list several possible second-line therapies: intrauterine tamponade, arterial embolization, vascular ligation (uterine artery ligation, hypogastric artery ligation), uterine compressive suture (B-Lynch), and hysterectomy. Nevertheless, these guidelines do not specify which therapy should actually be used, in what order or in which circumstances, and none lists the specific indications for hysterectomy. These respondents’ choice of second-line therapies according to their usual practice and the clinical situation encountered explains the variations observed in our study. Embolization was the most common second-line treatment chosen. This result was consistent with the findings in the French cohort study by Kayem et al. [25]: the choice of embolization may however be dictated by the resources available in respondents' hospitals, for it requires the presence of a specific technical platform and specific specialist provider. French guidelines specify that embolization can be used for patients who are stable. In our study, however, 31.1% of obstetricians used embolization in vignette 1, step 3, despite the patient’s hypotension and active bleeding.

All four sets of guidelines specify that other members of the team must be called, in particular, senior staff members, with appropriate levels of expertise. However, in our study, only 52.9% obstetricians chose to alert an additional obstetrician in vignette 1 and only 29.4% in vignette 2. In their French cohort study of women with PPH, Driessen et al. showed that a call for additional assistance from a senior obstetrician occurring more than 10 minutes after the PPH diagnosis was independently associated with severity (adjusted OR 1.61, 95% CI 1.23–2.12) [35]. Another factor independently associated with PPH severity was oxytocin administration more than 10 minutes after PPH diagnosis (adjusted OR 1.38, 95% CI 1.03–1.85). Our study showed that obstetricians would delay administration of this hormone; 82.3% would not administer it until more than 15 minutes after diagnosis in vignette 1. Our results from obstetricians are similar to those we obtained from midwives with the same clinical vignettes [31]. Specifically, we also observed a delay in the administration of uterotonic treatment: 35% and 55% of midwives would not administer oxytocin until after step 1 (the first 15 minutes after diagnosis) in vignette 1 and vignette 2 respectively, 33% and 60% would not administer prostaglandin in the first 30 minutes in vignette 1 and vignette 2 respectively. These delays were also more pronounced for vignette 2.

Observational studies confirm these findings from clinical vignettes of delay in treatment and inadequate management of PPH. Variations described in observational studies are likely related to the diversity of situations encountered. We demonstrated that the variations were related to the situation encountered but also to the obstetrician individually. We chose two very different situations (vignette 1 and vignette 2) to verify that variations in practices between obstetricians dealing with the same situation exist, regardless of that situation. We found that management was more aggressive for vignette 1 (typical immediate severe PPH) than for vignette 2 (gradual severe PPH) and that the delay in step 3 was greater in vignette 2 although the total blood loss was similar (1.2 L). The specific clinical situation involved may thus partly explain variations in practices. Respondents probably underestimated the severity of vignette 2. Gradual PPH with a constant trickle of blood is more complex to diagnose and manage; it may also generate less stress. The apparently milder severity of PPH may partly explain a portion of the delay in action. Some of these variations are thus related to the clinical situation encountered.

### Strengths and limitations of the study

The similarity of our results to those of previous studies based on other methods [19,35] shows that clinical vignettes are a simple, inexpensive tool producing results at least as valid as more complicated methods for evaluating the quality of care and variations in practice. This method enabled us to describe important variations at the individual level, both between obstetricians and for two different situations managed by the same obstetrician. Few previous studies have shown variations in PPH management at the individual level [31].

This study has also some limitations. Professionals respond to case-vignettes theoretically, developing plans and intentions, but these are not actual practice. A vignette cannot instill the urgency or engender the stress induced by a real woman with a real PPH. Nor can it adequately translate the necessary multidisciplinary approach. This theoretical approach, based on reflection rather than action, is at greater risk of social desirability bias and may thus result in overestimates of guideline adherence and appropriate management. Similarly, evaluating practices by multiple-choice rather than open-ended questions also tends to overestimate participant performance [36]. Moreover, the available facilities at the unit where the obstetricians currently work may have influenced responses. However, the lack of details on resources available at responders' hospitals means it is difficult to ascertain whether responses for some treatments relate to theoretical practice in general, or to the constraints of local resources. Nonetheless, second-line therapy choices varied widely, and management was often delayed. Selection bias occurred in the selection and inclusion of maternity units and obstetricians and was aggravated by the low response rate. Public maternity units and senior obstetricians (>4 years of experience) were over-represented. This bias may limit the generalization of our results. The limited number of obstetricians per maternity unit (2 obstetricians per maternity on average, only one maternity with 5 obstetricians) did not allow us to calculate a center effect. Lastly, the obstetricians who agreed to participate were probably those who most interested in the topic and in improving the quality of their practice. They were thus more likely to be able to respond correctly to this theoretical exercise, although their results in real life might not necessarily be any better.

## Conclusion

Case vignettes demonstrated variations in PPH management between obstetricians and in different situations of PPH; they also demonstrated inadequate management. Research is required to develop and then evaluate methods to improve the dissemination of guidelines for better adherence and more effective practices. Protocols or procedures are necessary in all maternity units to reduce the variations in practices that may explain part of the delay in management that results in PPH-related maternal mortality and morbidity.

## Supporting information

S1 File Vignette 1. (PDF)

S2 File Vignette2. (PDF)

S1 Table Database of obstetricians responses. (XLSX)
